# An Online Survey for Pharmacoepidemiological Investigation (Survey of Non-Medical Use of Prescription Drugs Program): Validation Study

**DOI:** 10.2196/15830

**Published:** 2019-10-25

**Authors:** Joshua Curtis Black, Karilynn Rockhill, Alyssa Forber, Elise Amioka, K Patrick May, Colleen M Haynes, Nabarun Dasgupta, Richard C Dart

**Affiliations:** 1 Rocky Mountain Poison & Drug Center Denver Health and Hospital Authority Denver, CO United States; 2 Injury Prevention Research Center University of North Carolina at Chapel Hill Chapel Hill, NC United States

**Keywords:** nonprobability methods, general population survey, drug abuse, calibration weights

## Abstract

**Background:**

In rapidly changing fields such as the study of drug use, the need for accurate and timely data is paramount to properly inform policy and intervention decisions. Trends in drug use can change rapidly by month, and using study designs with flexible modules could present advantages. Timely data from online panels can inform proactive interventions against emerging trends, leading to a faster public response. However, threats to validity from using online panels must be addressed to create accurate estimates.

**Objective:**

The objective of this study was to demonstrate a comprehensive methodological approach that optimizes a nonprobability, online opt-in sample to provide timely, accurate national estimates on prevalence of drug use.

**Methods:**

The Survey of Non-Medical Use of Prescription Drugs Program from the Researched Abuse, Diversion and Addiction Related Surveillance (RADARS) System is an online, cross-sectional survey on drug use in the United States, and several best practices were implemented. To optimize final estimates, two best practices were investigated in detail: exclusion of respondents showing careless or improbable responding patterns and calibration of weights. The approach in this work was to cumulatively implement each method, which improved key estimates during the third quarter 2018 survey launch. Cutoffs for five exclusion criteria were tested. Using a series of benchmarks, average relative bias and changes in bias were calculated for 33 different weighting variable combinations.

**Results:**

There were 148,274 invitations sent to panelists, with 40,021 who initiated the survey (26.99%). After eligibility assessment, 20.23% (29,998/148,274) of the completed questionnaires were available for analysis. A total of 0.52% (157/29,998) of respondents were excluded based on careless or improbable responses; however, these exclusions had larger impacts on lower volume drugs. Number of exclusions applied were negatively correlated to total dispensing volume by drug (Spearman ρ=–.88, *P*<.001). A weighting scheme including three demographic and two health characteristics reduced average relative bias by 31.2%. After weighting, estimates of drug use decreased, reflecting a weighted sample that had healthier benchmarks than the unweighted sample.

**Conclusions:**

Our study illustrates a new approach to using nonprobability online panels to achieve national prevalence estimates for drug abuse. We were able to overcome challenges with using nonprobability internet samples, including misclassification due to improbable responses. Final drug use and health estimates demonstrated concurrent validity to national probability-based drug use and health surveys. Inclusion of multiple best practices cumulatively improved the estimates generated. This method can bridge the information gap when there is a need for prompt, accurate national data.

## Introduction

Large governmental surveys, such as the National Survey on Drug Use and Health (NSDUH) in the United States, are used for nationwide drug use surveillance, offering researchers substantial statistical power for subgroup analyses, questionnaire consistency over decades, comprehensive and validated questionnaires, and probability-based geographic sampling for nationally representative estimates. However, these types of surveys cost millions of dollars a year to conduct, require training of field agents, and have a 2-year lag for data publication [1].

Trends in drug use can change rapidly by month, and using study designs with flexible modules could present advantages. Timely data can inform proactive interventions against emerging trends, leading to a faster public response. Large population surveys have used computer-assisted interviewing [2-4] with increased accuracy of self-reported socially stigmatized behaviors [5,6]. Internet-based questionnaires are an extension of computer-assisted interviewing, albeit with additional sampling and validity concerns, but van Gelder et al [7] have specifically suggested that illicit drug use may be a use case for internet-based questionnaires in epidemiology [7].

The use of online panels for public health research has grown in recent years [8-11]. Survey panels are groups of individuals who opt in to take surveys for modest compensation on a wide array of topics, maintained by commercial panel-access vendors. The sampling frame is theoretically suitable, since 90% of US adults use the internet [12]. Beyond efficient and rapid recruitment, panels offer superior anonymity and reductions in social desirability bias compared with in-person interviews [8,9].

However, threats to validity unique to internet surveys require removing careless or improbable responses [13], calibrating sample representativeness [14], preventing missing data [15], and addressing low response rates [16]. Crucially, representativeness of the sample to the target population requires methodological development since straightforward approaches, like poststratification demographic weighting, are insufficient [17].

This paper describes the development of a comprehensive methodology that addresses threats to validity of using survey panels for national drug use estimates. The approach encompasses mobile-friendly interface, skip logic [18], response randomization [19], careless/improbable response exclusions [13,20], and calibration weighting [21,22]. External validity was assessed compared with three probability-based national surveys. To our knowledge, this is the first use of online panel data incorporating multiple best practices to produce nationally valid estimates regarding drug use.

## Methods

### Survey Overview

The Researched Abuse, Diversion, and Addiction-Related Surveillance (RADARS) System comprises multiple data sources that characterize and monitor drug use [23]. The goal of the Survey of Non-Medical Use of Prescription Drugs (NMURx) Program described here is to provide accurate and timely estimates of prescription drug nonmedical use (NMU) and associated motivations and behaviors in the adult general US population. The NMURx Program employs a cross-sectional, opt-in online self-administered questionnaire drawn from a commercial survey panel. Respondents’ personal information is kept confidential by the survey administrators; personally identifiable information is not collected on the questionnaire, and information held by the survey administrators is not available to the researchers. Following best practices for implementation of online questionnaires [19], three methodological practices are described: reduction of order effect bias by randomization of question order, exclusion criteria based on careless/improbable responses, and calibration weighting for generalizability.

### Questionnaire Development

The main body of the questionnaire covered motivations and behaviors surrounding prescription drug use of four prescription drug classes (pain relievers, sedatives, stimulants, and cannabinoids), documenting lifetime and last 12-month NMU. NMU of prescription drugs was defined as use “in a way not directed by your health care provider.” Examples of the questionnaire questions can be found in Multimedia Appendix 1, Section A, followed by a list of drug classes and substances included in the questionnaire (Multimedia Appendix 1, Section B). Additional sections in the survey (some not included in this analysis) are: demographics, Drug Abuse Screening Test (DAST-10) measuring severity of problematic drug use [24], motivations and drug use behavior (eg, reasons for use, route of administration, source of acquisition), and health status (eg, substance use disorder treatment history, mental health disorders). Skip logic was used to minimize the number of questions a respondent was required to answer, with focus on preventing motivated underreporting [18]. Due to the large number of drugs included on the questionnaire and the follow-up questions on behaviors, motivated underreporting was of particular concern. The Checklist for Reporting Results of Internet E-Surveys (CHERRIES) is provided for further details on survey development and implementation (Multimedia Appendix 1, Section C).

Two strategies were used to reduce order effect bias. First, the order of drug classes was randomized, followed by order of substances within each drug class. Block randomization kept together similar substances (eg, all pain relievers), with the order consistently maintained throughout the survey sections. Second, respondents were forced to provide product-specific answers for last 12-month NMU of specific drug products that had been endorsed at the class level. This was intended to improve internal validity and further reduce order effect bias [19].

### Participant Sampling

The survey was open from September 28 through November 21, 2018. To be eligible for the survey, respondents must have been aged 18 years or older, and they must not have completed a NMURx Program survey in the same calendar year, excluding a small number of potential respondents who participated in pilot surveys. The panel company recruited panelists from the US population without specific consideration for the NMURx Program survey; selection into the panel was nonprobability-based and was a self-selected population of people who take surveys for compensation. The panel company employs evolving proprietary techniques to ensure panelists are providing reliable responses, with inactive or fraudulent accounts culled regularly. The panel company calculated each panelist’s expected response likelihood based on recent activity. These probabilities were used to select a random sample of panelists expected to yield 30,000 completed questionnaires. The email invitation did not include information about the survey topic to minimize selection bias; the topic was provided once a panelist opened the link during the consenting process.

To reduce the possibility of extreme analytical weights, 8-stratum sampling quotas were devised, proportionally based on the adult residential population from the American Community Survey (ACS) [25], stratified by male/female gender for four Census regions (Northeast, Midwest, South, and West). Based on pilot experience, each stratum was allowed a –25% and +10% range of acceptable number of surveys. Once all quotas reached their minimum and at least 30,000 questionnaires were completed, the survey link was closed. Survey respondents were compensated roughly US $1.

### Exclusion Criteria Assessment

Due to programmable internal data consistency checks, an outstanding concern after survey administration was the identification of completed questionnaires exhibiting careless or improbable response patterns (eg, endorsing all drugs at biologically improbable frequency). Methods used to exclude responses were adapted [13,20] to generate exclusion criteria that were validated against other questionnaire elements.

Based on previous implementation experience and literature review of consumer product surveys, seven different metrics were investigated as possible exclusion criterion using multiple hurdles [13], and four were chosen based on performance: (1) consecutive positive use endorsements of up to 42 prescription drugs based on the LongString method [20], (2) alternating patterns of yes/no endorsement of prescription drugs based on the even-odd consistency method [20] with Pearson correlations no more negative than –0.6, (3) alternating patterns of illicitly manufactured drugs with Pearson correlations no more negative than –0.8 for fewer drugs, and (4) total number of specific products endorsed for NMU in last 12 months via modified outlier analysis (out of 298 possible, most respondents only endorsed a handful) [13]. Completion time of 8 introductory questions in less than 16 seconds [13] did not provide additional discriminatory value (data not shown).

Since no gold standard was available for validation of the critical lifetime and 12-month prevalence questions, three internal consistency metrics were developed. Derived from other survey sections, these metrics provided biologically plausible support for responses to NMU: (1) survey response time for lifetime prescription drug use was a proxy for completion speed; (2) Mahalanobis distances were calculated on lifetime prescription, nonprescription, and illicitly manufactured drug use responses, representing deviance compared to the entire sample [13]; and (3) proportion of contradictory answers was calculated. For example, respondents were asked the time frame in which they had first initiated NMU and when they most recently nonmedically used, and the skip logic of the questionnaire allows for contradictory answers. Cut points were identified based on established theories (described in Results), visual inspection, and correlation coefficients [26].

To evaluate internal consistency, cut points were evaluated against demographics, drug use behaviors, and overall drug endorsements. To evaluate external consistency for careless responses, we compared relative endorsements to national opioid dispensing data from the US-based Longitudinal Patient Databases (IQVIA Inc), a standard source that provides estimates of dosage units dispensed in retail pharmacies. Since low-volume drugs should result in fewer endorsements, we hypothesized that careless responses would be roughly proportional to dispensing (using Spearman correlation), and excluded responses would account for a larger proportion of low-volume drugs.

### Calibration Weighting for National Estimates

**Figure 1:**
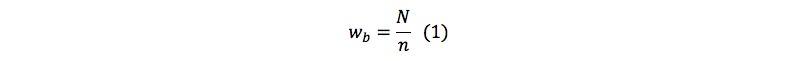
Base weight equation.

A calibration weighting scheme was developed to generate national estimates for the adult population. The goal of the weighting scheme was to reduce the bias in estimates resulting from the self-selection of survey panelists by forcing the distribution of our sample to look similar to national estimates across demographics and health-related variables. Generalized raking using auxiliary information with incomplete stratification was selected as the method of calibration weighting [21,22] because raking has been shown to be equivalent or superior to propensity score methods or sample matching in reducing bias [27,28]. Briefly, this method matches the marginal distributions of each variable in the sample to the marginal distribution from the population by iteratively adjusting the base weights. The base weight (w_b_) was calculated where N is the adult population in 2017 (N=252,063,800) and n is the sample size (Figure 1).

The analytical weight was calculated using established procedures [29]. Maximum tolerance was 0.1 percentage points; convergence occurred at a tolerance of 0.1 weighted frequency. The national marginal values were obtained from two 2017 probability-based national surveys, ACS and National Health Interview Survey (NHIS) [2,25].

Eight potential weighting variables from ACS and NHIS were selected based on associations with three lifetime measures also appearing in our questionnaire: any illicit drug use, any prescription pain reliever NMU, and any prescription NMU. Three demographic variables (age, sex, Census region of residence) and two household characteristics (household income and number of people in the home) were derived from ACS. Three health-related characteristics (self-assessment of general health, limitations in daily activities, and smoking tobacco) came from NHIS. To match basic demographics of the adult population, the three demographic variables were included in every model. Raking was tested against all remaining combinations (33 possible schemes).

**Figure 2:**
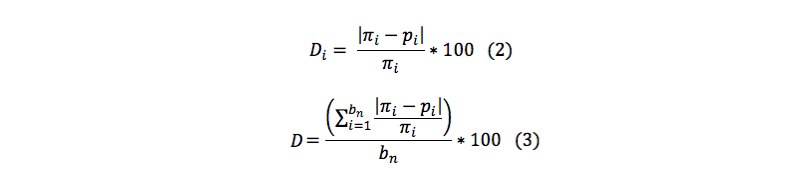
Absolute value of the relative difference and average relative difference equations.

To evaluate the 33 possible schemes, 26 benchmark national estimates were compared between the NMURx Program and four national surveys: ACS, NHIS, NSDUH, and National Health and Nutrition Examination Survey [3,4]. The absolute value of the relative difference (D_i_) for the i^th^ benchmark between the NMURx Program estimate (p_i_) and the national survey estimate (π_i_) were calculated (Figure 2, equation 2). These were averaged across the 26 benchmark estimates (Figure 2, equation 3, where b_n_ is the number of benchmarks).

The average relative difference in estimates across the weighting schemes for health-related benchmark estimates (overnight stay in a hospital, pain reliever use, illicit drug use, and alcohol use) compared with nonhealth-related benchmarks (race/ethnicity, marital status, education, employment, and insurance status) was evaluated. Final analytical weights represented the number of adults that a survey respondent would represent in the United States, generating national prevalence estimates, with 95% confidence intervals using variance estimation through Taylor series linearization [30].

### Ethics Review

The protocol and survey instrument were reviewed and approved by the Colorado Multiple Institutional Review Board; a certificate of exemption was granted on July 5, 2016.

## Results

### Participant Recruitment

**Figure 3:**
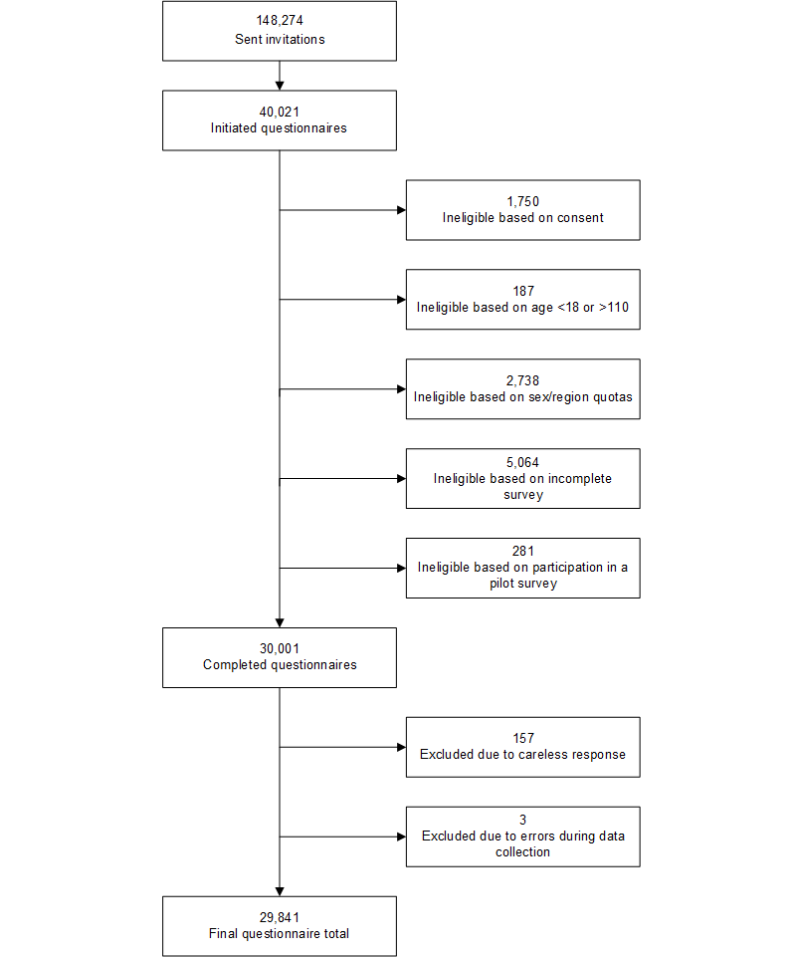
Flow diagram of respondents invited to the survey.

There were 148,274 invitations sent to panelists, and 40,021 (26.99%) people initiated the survey. After eligibility assessment, 74.96% (29,998/40,021) of the completed questionnaires were available for analysis (Figure 3). After careless responses were removed, the final participation rate was 20.13% (29,841/148,274). Out of 910 3-digit zip codes in the United States, 883 zip codes had at least one respondent. An order effect was present, and the likelihood of endorsement for individual active pharmaceutical ingredients was associated with the position in which the item was presented in their questionnaire (Multimedia Appendix 1, Section D).

### Evaluations of Exclusion Criteria

There were 157 respondents (0.52% of the sample) excluded based on careless or improbable responses (Multimedia Appendix 1, Section E). The final sample had a median completion time of 10 minutes, 40 seconds. For criterion 1, only 27 responses were identified, where over half of the 42 drug use questions were consecutively endorsed (Table 1). Consistent with recommendations [13], this cut point requires a respondent to endorse at least two separate drug classes in an unbroken string of “Yes” responses to be excluded. Criteria 2 and 3 for alternating responses resulted in 33 and 17 surveys being excluded, respectively. For criterion 4 on total drug endorsements, 91.01% (27,301/29,998) did not endorse any NMU in the last 12 months. Among those endorsing at least one product, median products endorsed was 3 (interquartile range 1 to 7) of 298 possible. Given the highly skewed distribution, visual inspection of Mahalanobis distance, question completion time, and contradictory answers were used to select 35 products in the last 12 months endorsed as a conservative cut point (Multimedia Appendix 1, Section F), resulting in 96 responses excluded. There was very little overlap across multiple criteria. There were 6 respondents who were identified by both criterion 1 and criterion 4. A total of 10 respondents were identified by both criterion 2 and criterion 3. Table 1 demonstrates that groups of respondents excluded by each criterion also demonstrated other behaviors indicative of careless response. Excluded respondents answered questions more quickly. Excluded respondents had greater mean Mahalanobis distances, and the proportion of excluded respondents who provided at least one contradictory answer on the survey was very different from the proportion of included respondents who provided contradictory answers (67/157, 42.7%, and 224/29,841, 0.75%, respectively). Excluded respondents more frequently reported being male, younger, and Hispanic compared with respondents who were not excluded, although statistical tests of differences were not conducted.

While a small proportion of responses were excluded (157/29,998, 0.52%), these exclusions had a larger impact on unweighted endorsements of lower volume active pharmaceutical ingredients (Table 2). The relative percentage decrease in responses endorsing NMU of opioid ingredients was negatively correlated to dispensing volume (Spearman ρ=–.88, *P*<.001), confirming our hypothesis that misclassification due to careless response would have a greater impact on low-volume products.

**Table 1:** Respondent characteristics of excluded and included respondents.

Characteristics		Criterion 1 (n=27)	Criterion 2 (n=33)	Criterion 3 (n=17)	Criterion 4 (n=96)	All excluded respondents^a^ (n=157)	Included respondents (n=29,841)
Male, n (%)		21 (77.78)	25 (75.76)	13 (76.47)	75 (78.13)	124 (78.98)	16,065 (53.84)
Age in years, median (IQR^b^)		34 (28, 37)	32 (28, 37)	35 (29, 43)	33 (28, 37)	33 (28, 37)	53 (35, 66)
**Race/ethnicity^c^, n (%)**							
	Hispanic/Latino(a)	8 (29.63)	12 (36.36)	8 (47.06)	26 (27.08)	47 (29.94)	2271 (7.61)
	White	19 (70.37)	26 (78.79)	13 (76.47)	72 (75.00)	116 (73.89)	24,946 (83.60)
	African American	—^d^	5 (15.15)	—^d^	17 (17.71)	27 (17.20)	2838 (9.51)
	Asian	—^d^	—^d^	—^d^	—^d^	6 (3.82)	1164 (3.90)
	Other	—^d^	—^d^	—^d^	—^d^	10 (6.37)	1649 (5.53)
Total time spent on prescription drug use question (seconds), median (IQR)		106.06 (80.98, 172.65)	73.26 (66.16, 89.73)	82.48 (66.16, 98.60)	97.77 (77.05, 143.40)	93.99 (73.22, 127.81)	116.31 (89.66, 159.53)
Mahalonbis distance, mean (SD)		18.89 (3.67)	28.45 (2.35)	29.28 (3.15)	24.15 (4.76)	24.53 (5.05)	6.60 (4.75)
Contradictory answers, n (%)		8 (29.63)	15 (45.45)	4 (23.53)	46 (47.92)	67 (42.68)	224 (0.75)

^a^Excluded respondents identified as any one of the four criteria established for careless response: LongString prescription drug use endorsements (criterion 1), even-odd consistency for prescription drug use (criterion 2), illicit drug use (criterion 3), or total product endorsement (criterion 4).

^b^IQR: interquartile range.

^c^Respondents may select multiple races, so percentage may not sum to 100.

^d^Cells with fewer than 5 respondents are suppressed for disclosure protections.

**Table 2:** Relative decrease in prescription drug nonmedical use endorsements after exclusions compared with drug availability.

Active pharmaceutical ingredient	No exclusions applied (n)	All exclusions applied (n)	Relative decrease (%)	Dispensing volume (dosage units dispensed)
Hydrocodone	905	855	5.52	4,570,914,825
Oxycodone	762	708	7.09	3,373,604,063
Tramadol	485	434	10.52	2,403,511,798
Codeine	742	687	7.41	1,986,127,916
Morphine	320	264	17.50	467,226,515
Hydromorphone	157	114	27.39	184,212,536
Tapentadol	83	53	36.14	48,107,328
Fentanyl	187	137	26.74	36,317,430
Oxymorphone	150	111	26.00	34,595,913
Dihydrocodeine	78	47	39.74	1,889,020

### Selection of Weighting Scheme

**Figure 4:**
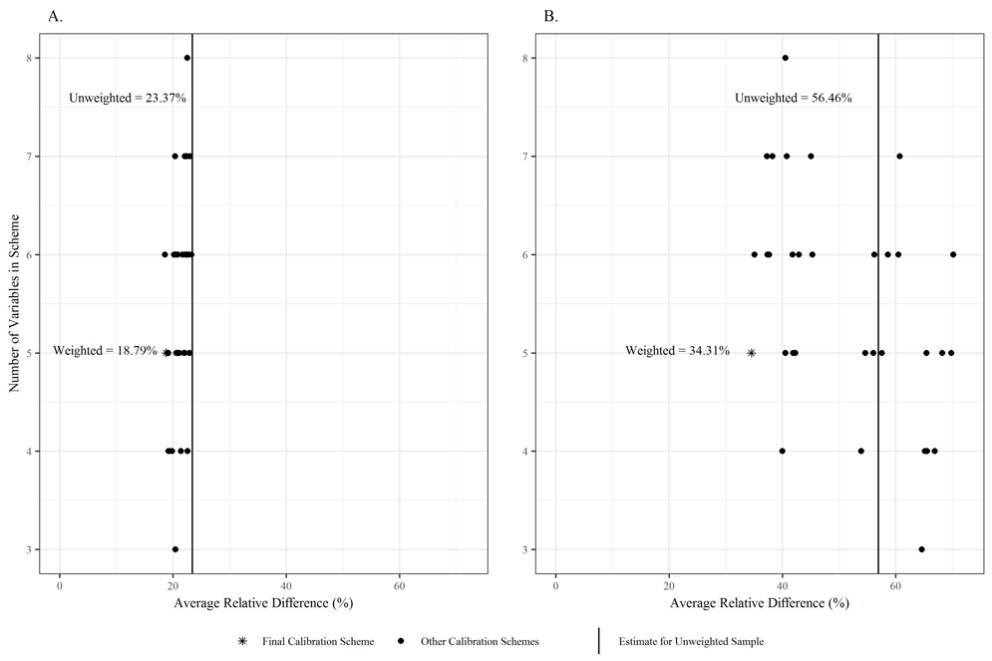
Average relative difference in nonhealth-related (A) and health-related (B) benchmarks with calibration weighting are shown for the 33 weighting schemes.

The remaining 29,841 surveys were used for calibration weighting. The unweighted NMURx Program data had an average relative difference of 36.1% compared with weighted estimates (Table 3). Across the 33 weighting schemes, the addition variables resulted in decreases in the average relative difference while relative standard error increased (Multimedia Appendix 1, Section G). Little variation in average relative difference was observed among nonhealth-related benchmarks; however, there were large changes in average difference among health-related benchmarks (Figure 4). Five-variable weighting schemes appeared to maximize reduction in relative difference and with minor increases in relative standard error, resulting in the selection including age, gender, region, limitation in daily activities, and tobacco use. This scheme had a 31.2% reduction in average relative difference compared with unweighted, resulting in 381 unique weights, none of which appeared extreme. The median weight was 7782.2 (interquartile range 4690.2 to 12,662.9).

**Table 3:** Relative difference in benchmark estimates.

Characteristic			Survey of Non-Medical Use of Prescription Drugs Program				National survey benchmark, % (95% CI)
			Unweighted		Weighted		
			Estimate, n (%)	Relative difference^a^ (%)	Estimate, % (95% CI)	Relative difference^a^ (%)	
**Nonhealth benchmarks**							
	**Race/ethnicity^b,c^**						
		Hispanic/Latino(a)	2271 (7.61)	–52.39	8.19 (7.82-8.56)	–48.78	15.99 (15.92-16.05)
		White	24,946 (83.60)	10.14	82.20 (81.69-82.71)	8.30	75.90 (75.83-75.97)
		African American	2838 (9.51)	–27.66	9.94 (9.55-10.34)	–24.35	13.15 (13.09-13.21)
		Asian	1164 (3.90)	–40.40	4.70 (4.41-4.99)	–28.16	6.54 (6.51-6.58)
		American Indian or Alaska Native	538 (1.80)	13.81	1.61 (1.45-1.77)	1.61	1.58 (1.56-1.60)
		Native Hawaiian or other Pacific Islander	108 (0.36)	–5.11	0.37 (0.29-0.45)	–3.31	0.38 (0.37-0.39)
		Other	1047 (3.51)	–29.90	3.88 (3.62-4.15)	–22.43	5.01 (4.97-5.04)
	**Marital status^c^**						
		Married	15,640 (52.41)	4.45	51.93 (51.29-52.57)	3.49	50.18 (50.10-50.26)
		Widowed	1928 (6.46)	7.60	5.17 (4.92-5.43)	–13.82	6.00 (5.97-6.04)
		Divorced	3969 (13.30)	16.08	11.50 (11.12-11.89)	0.40	11.46 (11.41-11.51)
		Separated^d^	576 (1.93)	—	1.76 (1.60-1.93)	—	2.02 (1.99-2.04)
		Never married	7728 (25.90)	–14.65	29.63 (29.02-30.24)	–2.35	30.34 (30.27-30.42)
	**Education^c^**						
		Less than high school	834 (2.79)	–76.84	2.69 (2.48-2.90)	–77.73	12.07 (12.01-12.12)
		High school graduate or GED^e^	5846 (19.59)	–29.12	18.65 (18.16-19.15)	–32.50	27.64 (27.57-27.71)
		Some college or associate’s degree	10,003 (33.52)	8.69	32.98 (32.38-33.58)	6.95	30.84 (30.77-30.91)
		Bachelor’s or higher degree or trade school^d^	13,158 (44.09)	N/A^f^	45.68 (45.04-46.31)	N/A	29.45 (29.38-29.52)
	**Employed last week^c^**						
		Yes	12,005 (40.23)	–34.89	45.77 (45.13-46.41)	–25.92	61.78 (61.70-61.86)
		No^d^	17,836 (59.77)	—	54.23 (53.59-54.87)	—	38.22 (38.14-38.30)
	**Private health insurance^g^**						
		Yes	19,007 (63.69)	–2.25	64.81 (64.20-65.42)	–0.54	65.16 (64.21-66.11)
		No^d^	10,834 (36.31)	—	35.19 (34.58-35.80)	—	34.84 (33.89-35.79)
**Health benchmarks**							
	**Overnight stay in hospital in last year^g^**						
		Yes	3609 (12.09)	43.13	9.65 (9.30-10.01)	14.26	8.45 (8.17-8.72)
		No^d^	26,232 (87.91)	—	90.35 (89.99-90.70)	—	91.55 (91.28-91.83)
	**Alcohol use in past 12 months^g^**						
		Yes	15,554 (52.12)	–3.88	52.10 (51.46-52.74)	–3.92	54.23 (53.15-55.31)
		No^d^	14,287 (47.88)	—	47.90 (47.26-48.54)	—	45.77 (44.69-46.85)
	**Pain reliever use^h^**						
		Lifetime use	18,287 (61.28)	–5.55	57.20 (56.57-57.84)	–11.83	64.90 (64.23-65.57)
		Past year use	9607 (32.19)	–9.54	27.32 (26.77-27.87)	–23.24	34.88 (34.21-35.55)
		Lifetime NMU^i^	3928 (13.16)	24.05	11.13 (10.74-11.51)	4.87	10.45 (10.05-10.84)
		Past year NMU	2313 (7.75)	76.58	6.18 (5.89-6.47)	40.78	4.06 (3.82-4.30)
		Past month NMU	1222 (4.10)	218.82	2.96 (2.77-3.15)	130.22	1.21 (1.07-1.34)
	**Illicit drug use^h^**						
		Lifetime use	6230 (20.88)	–16.70	17.53 (17.06-17.99)	–30.07	25.45 (24.85-26.05)
		Last year use	1756 (5.88)	54.63	4.74 (4.49-5.00)	24.61	4.08 (3.85-4.31)
		Last month use	970 (3.25)	111.67	2.45 (2.27-2.63)	59.31	1.68 (1.53-1.84)

^a^Relative difference was calculated using more significant figures than presented; due to rounding these results may appear different than calculating using estimates presented in this table.

^b^Multiple races may be selected so estimates may not sum to 100%.

^c^ACS: American Community Survey.

^d^Levels of estimates that were not included in average relative difference calculation.

^e^GED: General Educational Development test.

^f^Not applicable.

^g^NHIS: National Health Interview Survey.

^h^NSDUH: National Survey on Drug Use and Health.

^i^NMU: nonmedical use.

### External Validation Results

**Figure 5:**
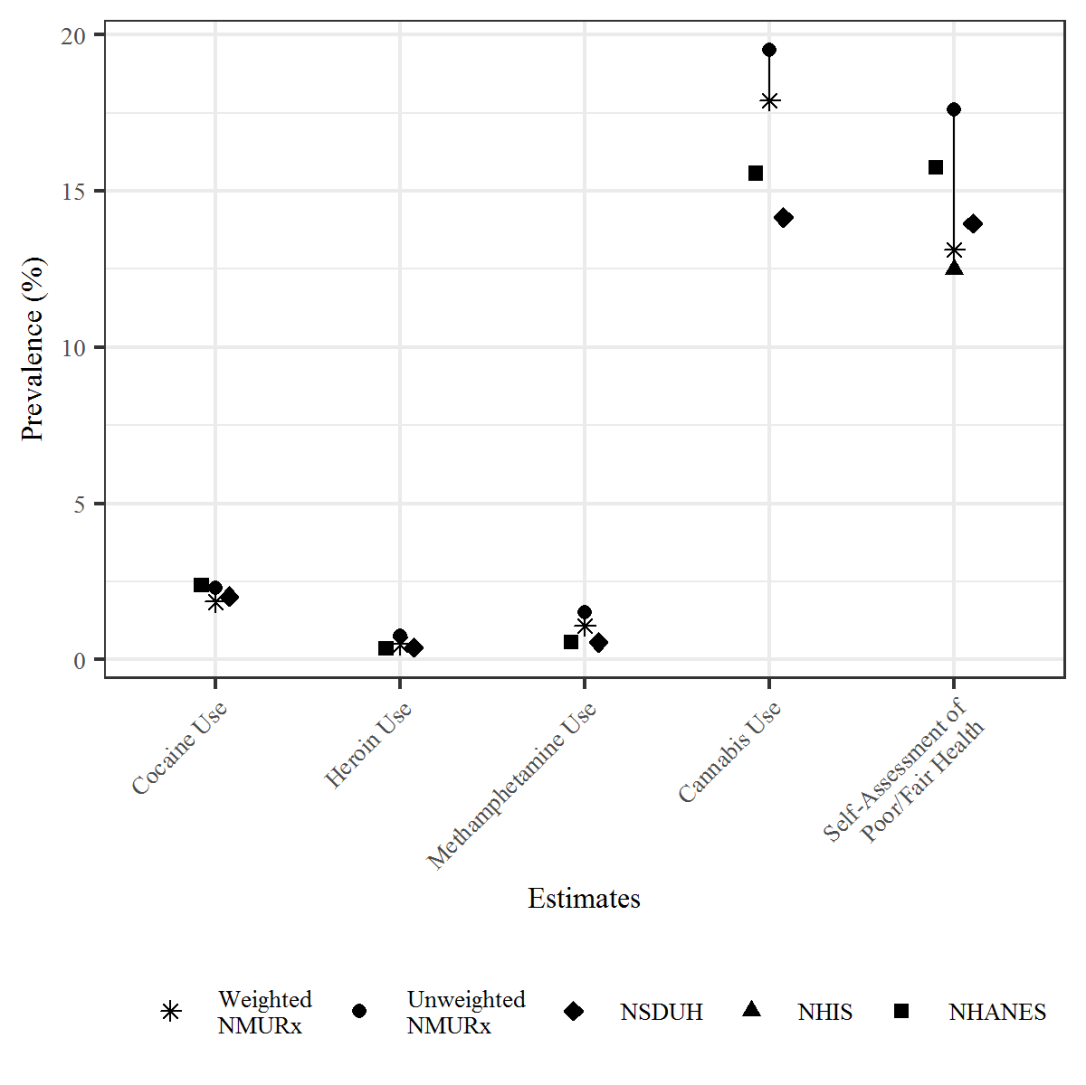
Comparison of estimates available across national surveys. NMURx: Survey of Non-Medical Use of Prescription; NSDUH: National Survey on Drug Use and Health; NHIS: National Health Interview Survey; NHANES: National Health and Nutrition Examination Survey.

After weighting, the NMURx Program estimates were closely aligned with the national benchmarks on demographic and other characteristics (Table 4). The unweighted sample had an overrepresentation of older adults, males, higher education, and lower household incomes, possibly a reflection of internet panel participants in general. The health profile of the sample was more similar to the national estimates after weighting. Proportions of good or excellent self-assessed health status and private insurance coverage increased while DAST-10 scores and the estimated proportion with chronic pain in the last year decreased. After weighting, NMU of any pain reliever decreased from 7.8% to 6.2%, sedatives decreased from 4.5% to 3.4%, and stimulants decreased from 2.4% to 2.0%. In addition, when comparing drug use and health indicator estimates across multiple probability surveys with similar questions, there was variation in estimates across probability-based national surveys, and NMURx Program weighted estimates were within similar ranges to the national surveys (Figure 5). Weighted estimates were closer to estimates from probability-based national surveys. Between national surveys, estimates of sex, age, and race were similar and confidence intervals generally overlapped; estimates of education varied slightly and in many cases confidence intervals didn’t overlap (Multimedia Appendix 1, Section H).

**Table 4:** Characteristics and national estimates before and after weighting.

Characteristics		NMURx^a^ Program unweighted n=29,841 n (%)	NMURx Program weighted n=252,063,800 % (95% CI)	ACS^b^ weighted n=252,155,280 % (95% CI)
**Age in years^c^**				
	18-24	2559 (8.58)	12.23 (11.75-12.71)	12.23 (12.18-12.29)
	25-34	4603 (15.43)	17.80 (17.28-18.31)	17.80 (17.73-17.86)
	35-44	4467 (14.97)	16.39 (15.90-16.88)	16.39 (16.33-16.45)
	45-54	4161 (13.94)	16.77 (16.27-17.28)	16.77 (16.71-16.83)
	55-64	5691 (19.07)	16.66 (16.22-17.10)	16.66 (16.61-16.72)
	65 or more	8360 (28.02)	20.15 (19.71-20.59)	20.15 (20.09-20.21)
**Sex^c^**				
	Male	16,065 (53.84)	48.67 (48.03-49.31)	48.67 (48.59-48.75)
	Female	13,776 (46.16)	51.33 (50.69-51.97)	51.33 (51.25-51.41)
**US Census region^c^**				
	Northeast	5219 (17.49)	17.74 (17.25-18.23)	17.74 (17.68-17.80)
	Midwest	6485 (21.73)	20.90 (20.39-21.41)	20.90 (20.83-20.96)
	South	11,485 (38.49)	37.74 (37.12-38.36)	37.74 (37.66-37.82)
	West	6652 (22.29)	23.62 (23.06-24.17)	23.62 (23.55-23.68)
**Annual household income**				
	<$25,000	6166 (20.66)	19.35 (18.84-19.85)	14.03 (13.98-14.09)
	$25,000-$49,999	8426 (28.24)	27.17 (26.61-27.74)	19.00 (18.94-19.07)
	$50,000-$74,999	6545 (21.93)	22.39 (21.86-22.93)	17.49 (17.43-17.55)
	$75,000-$99,999	4070 (13.64)	14.17 (13.72-14.62)	13.60 (13.54-13.65)
	$100,000 or more	4634 (15.53)	16.92 (16.43-17.41)	32.75 (32.67-32.82)
**DAST-10^d^ score**				
	None reported, 0	18,378 (61.59)	63.36 (62.74-63.98)	—^e^
	Low level, 1-2	9525 (31.92)	31.48 (30.89-32.08)	—
	Moderate level, 3-5	1396 (4.68)	3.83 (3.60-4.07)	—
	Substantial level, 6-8	428 (1.43)	1.04 (0.92-1.15)	—
	Severe level, 9-10	114 (0.38)	0.29 (0.23-0.35)	—
**Self-assessed health status**				
	Poor	754 (2.53)	1.63 (1.49-1.76)	—
	Fair	4496 (15.07)	11.48 (11.11-11.86)	—
	Good	11,313 (37.91)	37.10 (36.48-37.72)	—
	Very good	10,162 (34.05)	37.98 (37.35-38.61)	—
	Excellent	3116 (10.44)	11.81 (11.38-12.24)	—
**Chronic pain in last 12 months**				
	Yes	20,279 (67.96)	74.28 (73.75-74.81)	—
	No	9562 (32.04)	25.72 (25.19-26.25)	—

^a^NMURx: Survey of Non-Medical Use of Prescription Drugs Program.

^b^ACS: American Community Survey.

^c^These variables were used in weighting scheme, so marginal estimates will align with the ACS.

^d^DAST-10: 10-item Drug Abuse Screening Test.

^e^Not applicable.

## Discussion

### Principal Findings

While use of internet-based questionnaires for epidemiologic research has been previously described [31-33], our study illustrates a new approach to using nonprobability online panels to achieve national prevalence estimates for drug abuse. We were able to overcome challenges with using nonprobability internet samples [17,27,34,35], including misclassification due to careless or improbable responses. External validity of reweighted survey data demonstrated concurrent performance compared with large national probability surveys on demographics, health indicators, and drug use.

The value of internet samples is increasingly recognized [7,27,35,36], and our approach has strengths that may be relevant to drug use surveillance. Using calibration weights derived from two independent probability-sampled studies provided a hedge against overfitting [28,37]. The survey was fielded over the course of 8 weeks collecting at least 30,000 unique responses at a fraction of the cost of national probability samples. The entire process from fielding the survey to national estimates takes about 6 weeks. The ability to rapidly and inexpensively add new drugs to the survey is a considerable benefit against the background of the opioid crisis, which has evolved into its third phase, characterized by heroin-fentanyl deaths [38]. Beyond opioids, new drugs of abuse are being documented (eg, tianeptine, kratom) [39,40], while problematic drugs of the past are resurging (eg, methamphetamine, cocaine) [41]. Noncontrolled (nonscheduled) prescription drugs with abuse potential such as antidepressants [42], anticonvulsants [43], and novel psychoactive substances are not currently tracked on national probability surveys but could easily be added to online questionnaires. Emerging novel routes of administration (eg, intra-arterial injection), fluctuations in infectious disease risk factors, and uptake of harm reduction strategies could be queried in-depth. Our results also suggest that randomization is useful in mitigating order effects on surveys and skip logic is required to prevent motivated underreporting, neither of which is common practice yet on many national surveys. The method presented here cannot replace traditional probability-based surveys; in fact, it intentionally relies on those surveys to create optimized estimates. But this method can bridge the information gap when there is a need for prompt, accurate national data.

### Limitations

Ostensibly, the online-only setting creates the perception of anonymity between the respondent and researcher and reduces interviewer bias, but the role of the panel company as an intermediary and fears of data breaches may exert selection bias. There are putative gaps in the sampling frame since not all US adults use the internet. In terms of precision, 95% confidence intervals do not represent true 95% coverage probabilities because the exact selection probability from the sampling frame into the sample is not known, limiting statistical inferences within a purely frequentist context. Rather, the confidence intervals demonstrate precision of the estimates within the sampling framework, and inferences are useful when combined with an understanding of how the sample was obtained and weighted. Finally, a nonresponse adjustment was not included in this method. A drawback of using online panels is that information on nonresponding panelists is not available, and future extension of this work will be to obtain sufficient information in other ways to address this.

### Conclusions

We describe a practical approach to providing a timely perspective on drug abuse in the United States, with results obtained within 6 weeks of questionnaire deployment. The approach presented mitigates many valid concerns about the use of nonprobability internet panels and could be of use to other subject domains.

## Appendix

Multimedia Appendix 1 Supplemental methodological information and supporting results.

## Abbreviations

ACS: American Community Survey
CHERRIES: Checklist for Reporting Results of Internet E-Surveys
DAST-10: 10-item Drug Abuse Screening Test
NHIS: National Health Interview Survey
NMU: nonmedical use
NMURx: Survey of Non-Medical Use of Prescription Drugs
NSDUH: National Survey of Drug Use and Health
RADARS: Researched Abuse, Diversion and Addiction-Related Surveillance
