# Supplementary material for: An Online Survey for Pharmacoepidemiological Investigation (Survey of Non-Medical Use of Prescription Drugs Program): Validation Study
Source: J Med Internet Res. 2019 Oct 25;21(10):e15830. doi: 10.2196/15830 (PMC6914238; doi:10.2196/15830)
Supplement: Multimedia Appendix 1 [file jmir_v21i10e15830_app1.pdf]

Supplemental Material:

A. Question Wording Involved An all Analyses: The following is the exact wording of all questions used in the analyses. The format below is not indicative of the format seen on the device since format adjusts based on the device used.

First set of introductory questions completed by the respondent. Sex and three-digit zip code questions are used to determine quota status; the zip code provided by the respondent is mapped to state and census region. The total time spent on these eight questions was used in the exclusion criteria analyses. The questions regarding combined household income, number of people in the home, self-assessed health status, limitation in daily activities, and cigarette smoking were all contenders in the calibration weighting scheme.

What is your sex? Select one.
Male
Female

What is your age in years?
[Text Box] Please enter a whole number.

Please indicate the first three digits of the five digit ZIP code that you currently live in.
[Text Box] Enter a whole number.

What was your combined household income during the last 12 months? Select one.
Less than $25,000
Between $25,000 and $49,999
Between $50,000 and $74,999
Between $75,000 and $99,999
$100,000 or more

How many people are living or staying at your home including yourself and anyone who has nowhere else to stay? Please do not include anyone living somewhere else, such as a college student or armed forces member on deployment.

[Text Box] Enter a whole number.

Would you say your health in general is excellent, very good, good, fair, or poor? Drag the slider to answer the question.

Poor	*Fair*	*Good*	*Very Good*	*Excellent*

Are you currently limited in your usual activities in any way due to a medical condition? Select one.

Yes
No

Do you NOW smoke cigarettes every day, some days, or not at all? Please answer "Not at all" if you have smoked fewer than 100 cigarettes in your life. Select one.

Every day
Some days
Not at all
Don't know

Lifetime use of prescription drugs questions across the 42 active pharmaceutical ingredients were used in the following analyses: randomization, exclusion criteria. The total times spent on these questions were used as an internal validation measure for the exclusion criteria. The list of drug classes and active pharmaceutical ingredients were presented in a random order where the order of drug class is randomized, then the order of all active pharmaceutical ingredients within the drug class are randomized.

Have you ever used the prescription ***<drug group>*** *below? Select yes or no.*

<Scrolling text:> ***<Active pharmaceutical ingredient>*** *such as <products>*

Yes	*No*

The prompting text for non-medical use (NMU) was used as a prompting screen for all drug classes

We are interested in whether you have used any of these prescription medications ***in a way not directed by your healthcare provider****, even if just once or just a little. Examples include:*

- *You used it without a prescription or with someone else's prescription*
- *You used it in greater amounts, more often, or for longer than directed*
- *You used it for the experience or feeling it caused*
- *You changed it before use by crushing, chewing, dissolving, or heating it*

The last 12 month NMU of drugs is asked for each product and is used in the following analyses: exclusion criteria and endorsement comparison to volume of drug dispensed.

Have you used the prescription ***<drug group>*** *below in a way not directed by your healthcare provider in the last 12 months? below? Select yes or no.*

<Scrolling text:> ***<Active pharmaceutical ingredient>*** *such as <products>*

Yes	*No*

The questions regarding most recent NMU and first time for NMU allow the respondent to provide contradictory answers. The proportion of respondents who contradict themselves on the survey at least once was used in the internal validation measure for the exclusion criteria. A contradictory answer is defined as 1) answering the most recent use was 3 to 12 months ago, but answering the first time as within the last 90 days, or 2) answering the first use was 3 to 12 months ago, but answering the most recent use as longer than 12 months ago.

*When did you use <**drug**> in a way not directed by your healthcare provider…*
Drag and drop to an answer below.

…the most recent time?

In the last 90 days	*3 to 12 months ago*

*When did you use <**drug**> in a way not directed by your healthcare provider… Drag and drop to an answer below.*

…for the first time?

In the last 90 days	*3 to 12 months ago*	*More than 12 months ago*

Lifetime use of illicit drugs questions across the 19 substances were used in the exclusion criteria analysis.

*Have you **ever** used the drug below? Select yes or no.*

*<Scrolling text:> **<Illicit drug>** (<examples, slang>)*

Yes	*No*

B: List of drug classes and drug substances included in the questionnaire.

Pain Relievers	Sedatives	Stimulants	Cannabinoids	Illicit Drugs
Benzhydrocodone	Alprazolam	Amphetamine	Dronabinol	Alkyl Nitrate or Nitrous
Buprenorphine	Baclofen	Atomoxetine	Nabilone	Anabolic steroids not prescribed by a healthcare professional
Codeine	Chlordiazepoxide	Methlyphenidate		Cocaine Powder
Dihydrocodeine	Clobazem	Modafinil		Crack Cocaine
Eluxadoline	Clonazepam			GHB/GBL
Fentanyl	Chlorazepate			Heroin
Gabapentin	Diazepem			Ketamine
Hydrocodone	Estazolam			Kratom
Hydromorphone	Eszopiclone			LSD
Ketamine	Flurazem			MDMA
Methadone	Lorazepam			Mephedrone
Morphine	Midazolam			Mescaline
Oxycodone	Oxazepam			Methamphetamine
Oxymorphone	Quazepam			Non-pharmaceutical amphetamine
Pregabalin	Temazepam			Non-pharmaceutical fentanyl
Stufentanil	Triazolam			PCP or phencyclidine
Tapentadol	Zaleplon			Psilocybin or mushrooms
Tramadol	Zolpidem			Salvia
				Synthetic cannabinoid

				receptor agonists

C. CHERRIES Checklist for Reporting Results of Internet E-Surveys:

Item Category	Checklist Item	Explanation
**Design**	Describe survey design	The survey employs a cross-sectional design, an opt-in online self-administered questionnaire provided to panelists from a commercial survey panel. The general adult population in the United States is the target population.
**Institutional Review Board Approval**	IRB approval	The protocol and survey instrument were initially reviewed and approved by the Colorado Multiple Institutional Review Board; a certificate of exemption was granted on 5 July 2016.
	Informed consent	Panelists were offered an informed consent page that described the purpose of the survey as researching use of medications, tobacco, alcohol, drugs, and other health issues, the voluntary nature of the survey, and the privacy of the data collected.
	Data protection	No personally identifiable information was collected on the questionnaire nor was any such information available to the researchers.
**Development and pre-testing**	Development and testing	The questionnaire was tested in a separate test environment to ensure skip logic, question presentation, and technical aspects of the implementation.
**Recruitment process and description of the sample having access to the questionnaire**	Open vs closed survey	A closed design was used, where only those receiving an invitation from the panel company were allowed to participate.
	Contact mode	Participants were sent an email indicating selection into the survey; no information

		regarding the survey purpose was indicated.
	Advertising the survey	The survey was not advertised.
**Survey Administration**	Web/email	The survey was posted on the panel company internal portal. Respondents could only access the survey via the portal or a link through the email invitation.
	Context	The survey was posted on the panel company portal and could only be accessed via the portal. The survey was taken on the respondents own device.
	Mandatory/voluntary	The survey was voluntary.
	Incentives	Modest incentives were offered in the form of redeemable points (valued at approximately $1USD at the time of survey completion).
	Time/Date	The survey was fielded from 28 September through 21 November, 2018.
	Randomization of items and questions	First, the order of drug classes was randomized, followed by order of substances within each drug class. Block randomization kept together similar substances (e.g., all pain relievers), with the order consistently maintained throughout following survey sections.
	Adaptive questioning	Skip logic was used to minimize the number of questions a respondent was required to answer, with focus on preventing motivated underreporting.
	Number of items	A single item was presented per page.
	Number of screens (pages)	The number of screens varies considerably, and depends on how many individual drugs a respondent endorses.

	Completeness check	Each question that requires an answer will prompt the respondent before they can proceed past the page or a continue button is not available until a suitable answer is provided. Therefore, all questionnaires received pass a completeness check.
	Review step	Respondents were not allowed to go back and change/review prior answers.
**Response Rates**	Unique site visitor	Unique visitors are managed by the panel company so that individuals are only allowed a single login. De-duplication and fraudulent account deletion are handled by the panel company.
	View Rate	View rate was not captured.
	Recruitment rate	Recruitment rate was not captured.
	Completion rate	A total of 40,034 respondents started the survey with 30,002 completing the survey. This resulted in a 74.9% completion rate.
**Preventing multiple entries from the same individual**	Cookies used	Cookies were not used.
	IP check	IP checks were made by the panel company to ensure panelists are unique. No survey-specific IP check was conducted.
	Log file analysis	Log file analysis was not conducted.
	Registration	Registrations were handled by the panel company so that no panelist could take the survey a second time. Once completed, the survey link for each panelist was no longer active.
**Analysis**	Handling of incomplete questionnaires	Incomplete questionnaires were excluded.
	Questionnaires submitted with an atypical timestamp	Timestamp exclusion was evaluated, but no respondents submitted a survey in less

		than the predefined time of 16 seconds for the first 8 questions.
	Statistical correction	This manuscript describes a method for selection of a weighting scheme for statistical correction.

D: Results of Randomization. The percent of respondents who endorsed lifetime drug use decreased as the position in which the drug was shown increases. Morphine endorsements decreased from 22.11% in questionnaires where it was in the first position to 15.59% in questionnaires where it was in the 18th position. Oxycodone endorsements decreased from 36.83% to 28.44% and oxymorphone endorsements decreased from 6.25% to 2.76% comparing the surveys where the API was seen in the first position to those with the API in the 18th position.

Supplementary Figure 1. Percent Endorsement of Lifetime Use of Each Drug by Randomized Position on Survey. The likelihood of each API had an equal probability of appearing at each position (1 through 18).

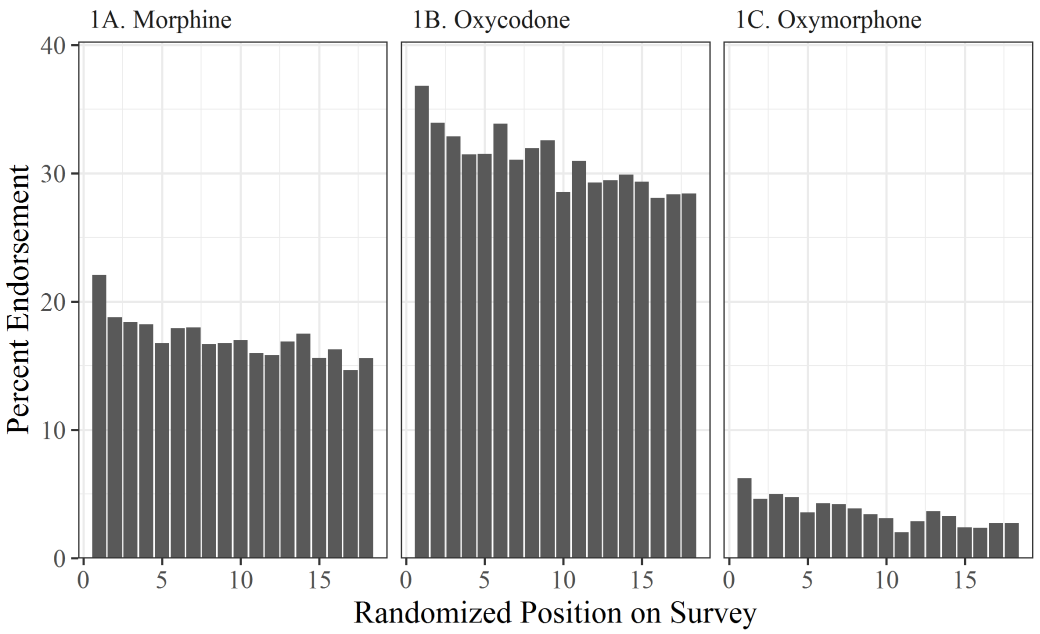

E: Distribution of Careless Response Patterns. Each histogram shows the distribution of exclusion metrics for all respondents. The number of respondents excluded is shown for each metric and is placed above respondents who would be excluded. The dotted line is the final chosen cut point threshold.

Supplementary Figure 2.

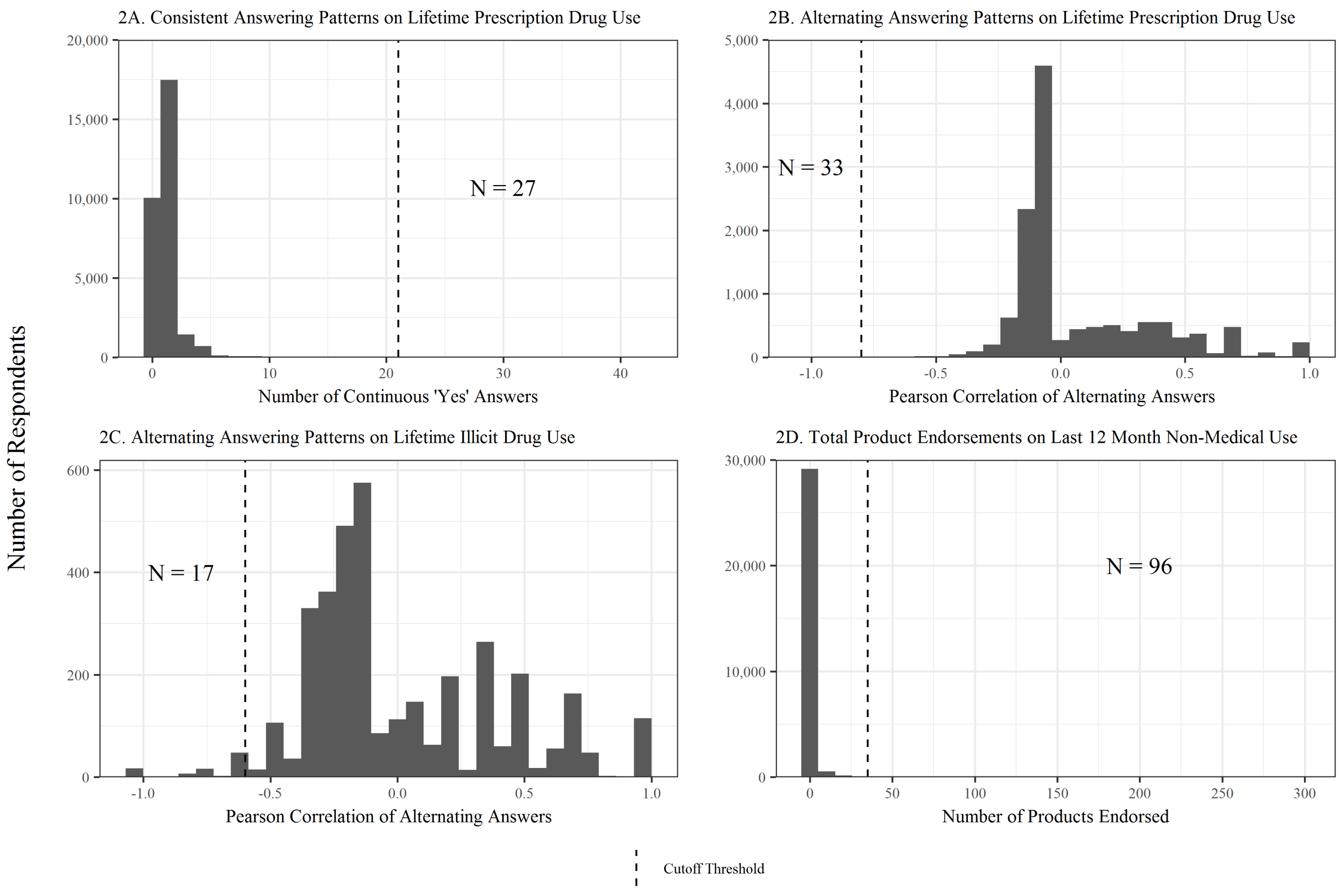

F. Exploratory Cut point Evaluation for Careless Response Criteria 4. For careless response Criterion 4, we evaluated the distribution of total number of products endorsed in the last 12 months NMU. This distribution was highly right skewed, and selection of the cut point requires further justification. Therefore, a series of cut point values were evaluated. At each cut point value, three internal validation metrics were calculated for included and excluded respondents. Plots of the internal validation metrics were examined for natural inflection points, though no clear cut point was observed. Therefore, a conservative cut point was selected where excluded respondents showed large differences in all metrics when compared to included respondents. The cut point was set to identify respondents who endorsed more than 35 products for NMU in the last 12 months, identifying 96 respondents. The x-axis is cut point values, with the excluded sample size based on that cut point shown in text next to each point. The y-axes are the three different internal metrics. The vertical line indicates the cut point selected.

Supplementary Figure 3.

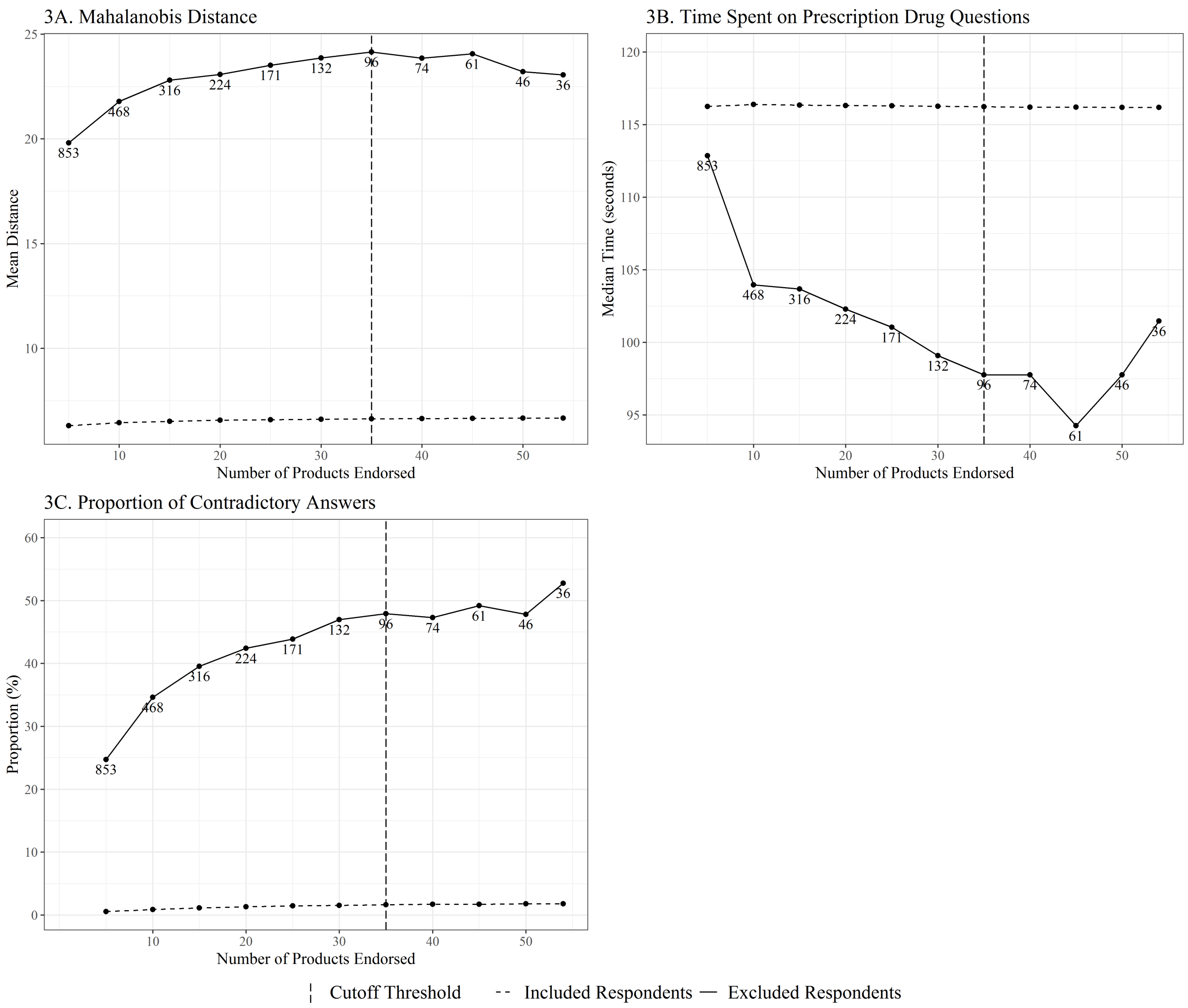

G. Calibration Weighting Scheme Model Benchmarked Estimates. The average relative difference (4A) and average relative standard error (4B) are shown for all 33 weighting schemes. The star shows the final chosen weighting scheme. The exact values for the unweighted estimates and final weighting scheme estimates are included.

Supplementary Figure 4.

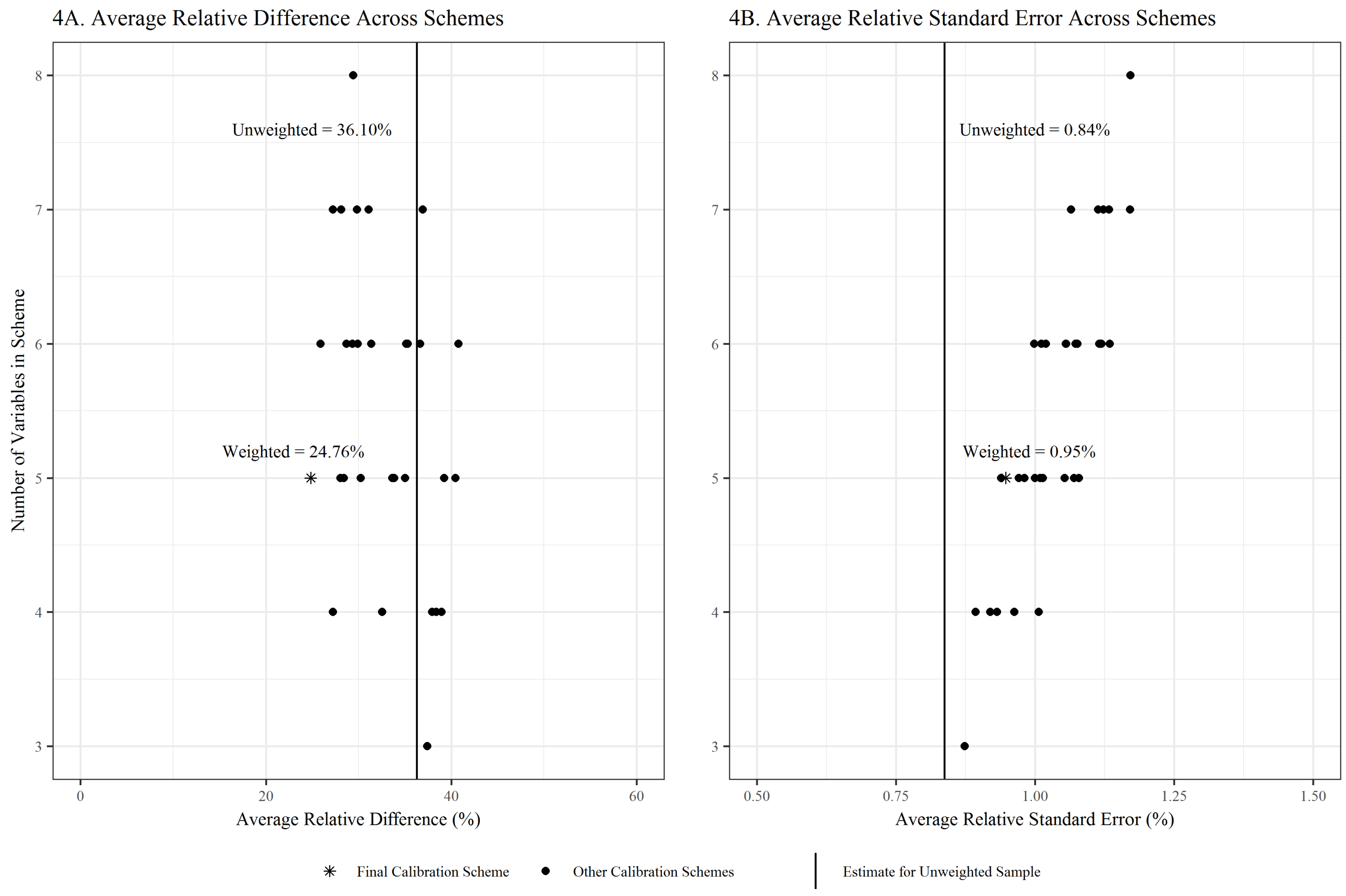

H. Comparison of Demographic Characteristics between Probability Surveys.

Respondent Characteristics	NSDUH 2016 % (95% CI)	NHIS 2017 % (95% CI)	NHANES 2015/2016 % (95% CI)	ACS 2017 % (95% CI)
**Male**	48.23 (47.53, 48.92)	48.24 (47.93, 48.55)	48.17 (46.42, 49.93)	48.67 (48.59, 48.75)
**Age Categories, (years)**				
18-24		12.05 (11.63, 12.47)	10.81 (9.85, 11.77)	12.23 (12.18, 12.29)
25-34		17.69 (17.24, 18.15)	18.79 (17.49, 20.09)	17.80 (17.73, 17.86)
35-44		16.33 (15.92, 16.74)	16.14 (14.89, 17.40)	16.39 (16.33, 16.45)
45-54		17.02 (16.63, 17.41)	18.00 (16.57, 19.44)	16.77 (16.71, 16.83)
55-64		16.84 (16.46, 17.22)	16.37 (14.94, 17.81)	16.66 (16.61, 16.72)
65+		20.07 (19.55, 20.59)	19.88 (18.50, 21.26)	20.15 (20.09, 20.21)
**Race/Ethnicity**				
Hispanic/ Latino(a)	15.74 (15.23, 16.26)	15.98 (14.66, 17.30)	15.60 (14.75, 16.45)	
Non-Hispanic White	64.39 (63.73, 65.06)	64.58 (62.97, 66.19)	63.35 (61.90, 64.80)	
Non-Hispanic Black	11.79 (11.37, 12.22)	12.24 (11.30, 13.18)	11.49 (10.78, 12.20)	
Non-Hispanic Asian	5.38 (5.04, 5.73)	6.06 (5.42, 6.69)	5.84 (5.38, 6.30)	
Other Race/Mixed Race	2.68 (2.49, 2.87)	1.15 (0.77, 1.52)	3.72 (3.08, 4.36)	
**Education**				
Less Than High School	12.89 (12.42, 13.37)	11.85 (11.26, 12.45)	14.88 (13.89, 15.86)	12.07 (12.01, 12.12)
High School Graduate or	25.28 (24.68, 25.88)	25.75 (25.11, 26.38)	21.45 (20.06, 22.85)	27.64 (27.57, 27.71)

GED				
Some College or Associate's Degree	30.88 (30.25, 31.51)	30.14 (29.48, 30.79)	32.29 (30.64, 33.94)	30.84 (30.77, 30.91)
Bachelor's or Higher Degree	30.94 (30.29, 31.60)	32.26 (31.23, 33.30)	31.38 (29.64, 33.12)	29.45 (29.38, 29.52)
